# Regulatory network remodeling and potential non-canonical associations of the transcription factor SP1 during inflammation-to-cancer transformation in esophageal squamous cell carcinoma

**DOI:** 10.3389/fimmu.2026.1919461

**Published:** 2026-10-16

**Authors:** Bin Du, Jie Zhang, Huimin Liu, Xiaoya Xing, Xinyue Wang, Pu Wang

**Affiliations:** 1 Shanxi Key Laboratory of Aging Mechanism Research and Translational Applications, Changzhi Medical College, Changzhi, China; 2 School of Basic Medical Sciences, Changzhi Medical College, Changzhi, China

**Keywords:** ESCC, inflammation-to-cancer transformation, potential non-canonical regulatory association, transcription factor Sp1, tumor immune microenvironment

## Abstract

**Background:**

Esophageal squamous cell carcinoma (ESCC) is closely linked to chronic inflammation, but the transcriptional mechanisms connecting epithelial inflammatory remodeling with malignant transformation remain incompletely understood.

**Methods:**

We integrated secondary analysis of published single-cell epithelial transition-state data, virtual SP1 perturbation, TCGA analyses, xCell immune infiltration analysis, TIDE-based exploratory immunotherapy prediction, Co-IP/MS, and a 4NQO-induced mouse model to investigate SP1-associated network remodeling during ESCC carcinogenesis.

**Results:**

SP1 regulatory importance increased across pairwise epithelial state comparisons. In adjacent normal epithelial cells, virtual SP1 overexpression affected genes linked to inflammatory response, NF-κB-related signaling, epithelial stress remodeling, and proliferation. SP1 expression correlated with immune infiltration features and immune checkpoint gene expression, and TIDE analysis suggested an association with predicted immunotherapy-related features. SP1-binding partners were enriched in RNA metabolism, RNA splicing, and nuclear protein-complex annotations, suggesting potential non-canonical regulatory associations that require further experimental validation.

**Conclusions:**

SP1 may represent a progressively enhanced transcriptional hub during inflammation-associated ESCC carcinogenesis, with state-dependent network remodeling spanning epithelial inflammatory stress, malignant transformation, and tumor immune features.

## 1 Introduction

Esophageal cancer is one of the gastrointestinal malignancies with a substantial global disease burden, and ESCC is the predominant histological type. Epidemiological data indicate that esophageal cancer ranks as the ninth most common malignancy worldwide and the sixth leading cause of cancer-related death (1). In 2020, there were approximately 604,100 new cases and 544,100 deaths globally, with squamous cell carcinoma accounting for approximately 85% of these cases. The incidence of ESCC exhibits marked geographic variation, with particularly high rates in regions such as East Asia and Eastern Africa, suggesting that its development may be closely associated with specific environmental exposures, lifestyle factors, and genetic susceptibility (2). Despite continuous advances in endoscopic diagnosis, and therapy, the overall prognosis for patients with ESCC remains unsatisfactory, and the five-year survival rate remains low (1). Elucidating the early molecular mechanisms underlying the transformation from normal epithelium to malignancy in ESCC is of great significance for the identification of early diagnostic biomarkers and interventional targets.

The development of ESCC arises from the interplay of environmental exposures, chronic injury, inflammatory responses, and genetic/epigenetic abnormalities. Tobacco smoking and alcohol consumption are among the risk factors with the most robust evidence; systematic reviews and meta-analyses have shown that current smokers have a significantly elevated relative risk of developing ESCC, and heavy drinkers also exhibit a markedly increased risk of the disease (3). The consumption of excessively hot food or hot beverages is also considered to be associated with an increased risk of ESCC (4). Furthermore, low socioeconomic status, nutritional deficiencies, unhealthy dietary patterns, and exposure to chemical carcinogens such as nitrosamines have also been implicated in the development and progression of ESCC (5, 6). Although these risk factors are of diverse origins, they can all lead to persistent irritation, repeated injury, and chronic inflammation of the esophageal epithelium, thereby creating conditions that facilitate the evolution from normal epithelium to precancerous lesions and ultimately to invasive carcinoma.

From a pathological perspective, the development of ESCC is generally considered to follow a sequential process that progresses from normal squamous epithelium through basal cell hyperplasia, intraepithelial neoplasia or dysplasia, and carcinoma *in situ*, ultimately culminating in invasive squamous cell carcinoma (7). This process indicates that ESCC does not arise abruptly but rather results from the gradual acquisition of abnormal proliferation, anti-apoptotic capacity, genomic instability, and invasive potential by esophageal epithelial cells under sustained microenvironmental stress (8).

Chronic inflammation is recognized as a crucial bridge linking external risk factors to epithelial malignant transformation. Prolonged exposure to tobacco, alcohol, thermal stimulation, or chemical carcinogens can induce esophageal epithelial injury and local inflammatory responses, accompanied by inflammatory-cell infiltration and release of cytokines, chemokines, and growth factors (9). These mediators can activate epithelial pro-survival and pro-proliferative signaling, enhance cell-cycle progression, inhibit apoptosis, and promote tissue remodeling (10, 11). For instance, LAMC1 can be upregulated through TGF-beta-related signaling and, in synergy with SAMD4 and SP1, promote ESCC progression; concurrently, it can induce inflammatory cancer-associated fibroblast formation through the CXCL1/CXCR2/pSTAT3 axis (12). PAR1 and PAR4 have also been reported to regulate ESCC growth and metastasis through STAT3 and NF-κB signaling (13). These findings suggest that inflammatory signaling may participate in reshaping epithelial cell fate, although the transcriptional regulatory mechanisms linking chronic inflammation to malignant transformation remain incompletely defined.

Single-cell transcriptomic studies have provided an opportunity to dissect multistage ESCC evolution and have revealed immune microenvironmental changes from transient activation to immunosuppression during chronic inflammation (14). These studies also indicate that transcription factors can act as regulatory hubs linking extracellular stress signals to epithelial cell-state transitions. In inflammation-associated tumorigenesis, STAT1 and STAT3 have been implicated in immune signaling, cell survival, and tumor progression (15, 16). Hippo-YAP signaling can also contribute to ESCC progression by regulating immune evasion-related molecules (17). However, most existing work has focused on cell-population composition and signaling-pathway annotation, whereas how epithelial transcriptional regulatory networks are remodeled during the transition from inflamed epithelium to malignant epithelium remains less clear.

SP1 is a ubiquitously expressed C2H2 zinc-finger transcription factor that binds GC-rich promoter elements and regulates genes involved in basal transcription, epithelial homeostasis, proliferation, differentiation, apoptosis, and stress responses (18). In cancer, SP1 has been widely studied as a context-dependent transcriptional regulator rather than a single pathway-specific oncogene (19). In tumor immunity, SP1 has been linked to immune checkpoint regulation, including PD-L1-, VISTA-, and B7-H3-related transcriptional programs in different cancer contexts (20–22). In ESCC, SP1 has been implicated in TGF-beta-related transcriptional regulation, including cooperation with SAMD4 in LAMC1 upregulation and inflammatory cancer-associated fibroblast formation (12). However, whether SP1-associated regulatory programs are dynamically remodeled during the transition from inflamed esophageal epithelium to ESCC remains insufficiently defined.

Against this background, this study focuses on the interplay between the inflammatory microenvironment and epithelial transcriptional regulatory remodeling during the transition from normal esophageal epithelium to ESCC. We specifically examined SP1 because of its known roles in epithelial biology, stress responses, tumor progression, and immune-related regulation. By integrating pathological progression, published single-cell transition-state data, virtual perturbation, TCGA-based immune analysis, Co-IP/MS, and a 4NQO-induced mouse model, this study aimed to characterize state-dependent SP1-associated regulatory programs during ESCC inflammation-to-cancer transformation.

## 2 Methods

### 2.1 Identification of SP1-associated regulatory dynamics during esophageal inflammation-to-cancer transformation

To identify SP1-associated transcription factor dynamics during esophageal inflammation-to-cancer transformation, we performed a secondary analysis of the published 4NQO single-cell epithelial transition-state dataset by Yao et al. (14) (GSA accession: CRA002118). The EpiC1-EpiC6 epithelial transition states and stage-associated gene information were obtained from the processed data and supplementary files of the original study, without *de novo* clustering or redefinition of cell states.

Genes altered between pairwise epithelial states were subjected to functional clustering and transcription factor enrichment analysis using Metascape (23). For transcription factor enrichment, the TRRUST database implemented in Metascape was used to identify potential upstream transcriptional regulators. Enriched transcription factors were ranked according to enrichment significance, and the ranking of SP1 across the pairwise state comparisons was summarized to evaluate its changing regulatory importance during esophageal carcinogenesis. Pairwise comparisons were defined as EpiC2 versus EpiC1, EpiC3 versus EpiC2, EpiC4 versus EpiC3, EpiC5 versus EpiC3, and EpiC6 versus EpiC5. EpiC1-EpiC3 are present at every stage, EpiC5 emerges at hyperplasia and predominates in invasive carcinoma, EpiC6 is essentially confined to invasive carcinoma, and EpiC4 is a differentiation branch rather than a step of malignant progression; because the EpiC2 versus EpiC1 comparison yielded no significantly enriched transcription-factor cluster, the late malignant-progression transitions used for cross-validation were defined as EpiC3→EpiC5 and EpiC5→EpiC6. Mouse gene symbols were mapped to their human orthologues for annotation (Input as species: M. musculus; Analysis as species: H. sapiens), and genes without a recognized orthologue were not annotated. The mapping was used for annotation only; the expression values and cluster markers shown in the heatmaps are those measured in mouse cells.

### 2.2 4NQO-induced mouse model of esophageal cancer

Forty-eight 6-week-old male C57BL/6 mice were acclimated for one week and then randomized into a control group (n = 8) and five experimental groups (n = 8 per group). To induce esophageal carcinogenesis, mice in the experimental groups received 4-nitroquinoline 1-oxide (4NQO; N113910, Aladdin) in drinking water at a concentration of 100 μg/mL. The 4NQO-containing water was freshly prepared twice per week and administered continuously for 16 weeks, after which the mice were switched to regular drinking water. Mice were sacrificed by carbon dioxide asphyxiation at predetermined time points: 12, 20, 22, 24, and 26 weeks. The control group received regular drinking water throughout the entire experiment and was sacrificed at 26 weeks. All animal experiments were approved by the Ethics Committee of Changzhi Medical College (DW2025181).

### 2.3 Histopathology, SP1 immunohistochemistry, and H-score quantification

Mouse esophageal tissues were fixed, embedded in paraffin, sectioned, and subjected to hematoxylin and eosin staining and SP1(21962-1-AP, Proteintech, China) immunohistochemistry. SP1 protein expression was evaluated in epithelial cells across the pathological stages of the 4NQO-induced esophageal carcinogenesis model, including normal epithelium, inflammation, hyperplasia, dysplasia, carcinoma *in situ*, and invasive carcinoma. Nuclear SP1 staining was assessed semi-quantitatively using the A.H.-score (H-score) method. For each pathological stage, H-scores were obtained from three independent animals (one tissue sample per mouse; n = 3 per stage). For each sample, the score was calculated as 1 × the percentage of weakly positive cells + 2 × the percentage of moderately positive cells + 3 × the percentage of strongly positive cells, yielding a theoretical range of 0-300. The proportion of strongly positive epithelial cells was also quantified for each pathological stage. Each animal was assigned to a single pathological stage, and different animals were scored for each stage; the three H-score values per stage therefore represent independent biological replicates.

### 2.4 Human single-cell data processing and epithelial-cell annotation

Human single-cell RNA-sequencing data were obtained from the GEO database (GSE196756). The dataset was generated from ESCC patient-derived samples using the 10x Genomics platform and Illumina NovaSeq 6000 sequencing; samples GSM5900215, GSM5900218, GSM5900219, and GSM5900220 were included as described in the original dataset annotation. The expression matrices were processed using Seurat. Genes detected in fewer than 3 cells were removed, and cells with fewer than 200 detected genes were excluded. Cells with 200 to 2,000 detected genes and mitochondrial transcript content below 10% were retained for downstream analysis. The data were normalized using the LogNormalize method with a scale factor of 10,000, followed by logarithmic transformation and scaling with the ScaleData function. The top 2,000 variable genes were used for principal component analysis, and the first 20 principal components were retained for dimensionality reduction and clustering.

Cell-type annotation was performed using SingleR with the Human Primary Cell Atlas reference dataset. Epithelial cells were identified based on SingleR annotation and evaluated according to canonical epithelial markers, including EPCAM, KRT5, KRT14, KRT13, KRT15, and TP63. Cells showing predominant expression of immune, endothelial, or fibroblast markers, such as PTPRC, PECAM1/VWF, or COL1A1/DCN, were excluded from epithelial-cell-based perturbation analysis. No new human tissue collection, cell isolation, or primary culture was performed for this analysis; therefore, no additional ethical approval was required for the secondary analysis of publicly available de-identified single-cell data.

### 2.5 Virtual SP1 overexpression and knockout analysis

To simulate the regulatory effects of SP1 perturbation in epithelial cells, we used two computational frameworks: scTenifoldNet for virtual overexpression and scTenifoldKnk for virtual knockout (24). For virtual overexpression, scTenifoldNet constructs an artificial overexpression matrix from the original expression matrix by increasing SP1 expression while keeping the remaining expression profile structure comparable. For virtual knockout, scTenifoldKnk sets SP1 expression to zero and evaluates the resulting network-level perturbation. Both algorithms construct gene regulatory networks from single-cell transcriptomic data and compare network topology before and after perturbation. Z-scores, log2 fold changes, and adjusted P values were used to identify genes most affected by simulated SP1 overexpression or knockout.

For exploratory biological interpretation of virtual perturbation results, the top 100 genes ranked by perturbation effect were submitted to Metascape for functional enrichment analysis. This fixed-rank approach was used because some perturbation settings yielded too few FDR-significant genes for stable enrichment analysis; it was not used to define statistically significant perturbations. For cross-dataset overlap analysis, the TRRUST-SP1-supporting gene sets of the two late transitions of the corrected trajectory (EpiC3→EpiC5 and EpiC5→EpiC6) were compared with genes significantly affected by virtual SP1 overexpression or knockout (FDR < 0.05), and enrichment was assessed with the hypergeometric test, considering only genes assessable in the perturbation background. Immune-related enrichment terms were interpreted as epithelial-cell-intrinsic inflammation-associated gene programs because all virtual perturbation analyses were performed within epithelial cells rather than immune cells.

### 2.6 TCGA ESCC cohort and prognostic model construction

STAR-counts data and corresponding clinical information for ESCC patients were obtained from The Cancer Genome Atlas (TCGA). Raw counts were converted to transcripts per million (TPM) values and log2(TPM + 1)-transformed. ESCC samples with complete clinical records were retained for downstream analysis. Genes identified from the SP1 virtual perturbation analysis in ESCC epithelial cells were used as candidate variables for exploratory prognostic model construction.

Least absolute shrinkage and selection operator (LASSO) regression analysis was performed to identify prognosis-related SP1 perturbation genes and construct an exploratory risk-score model. The optimal penalty parameter was selected by cross-validation. This model was developed and evaluated within the same TCGA-ESCC cohort and has not been validated in an independent external cohort; all prognostic results are therefore exploratory. Patients were stratified into high-risk and low-risk groups according to the median risk score. Overall survival was compared using Kaplan-Meier survival analysis and the log-rank test. Time-dependent receiver operating characteristic curve analysis was used to evaluate the predictive performance of the model.

### 2.7 Pathway correlation analysis of SP1 in TCGA ESCC samples

Pathway activity scores were calculated using single-sample gene set enrichment analysis implemented in the GSVA R package based on the log2(TPM + 1)-transformed TCGA ESCC expression matrix. Spearman correlation analysis was used to evaluate associations between SP1 expression and pathway activity scores. Pathways significantly correlated with SP1 expression were summarized to characterize SP1-associated biological programs in ESCC.

### 2.8 Immune infiltration analysis using xCell

Immune infiltration in TCGA ESCC samples was estimated using the xCell algorithm based on log2(TPM + 1)-transformed expression profiles (25). xCell was used to infer enrichment scores for immune and stromal cell signatures, as well as immune score and microenvironment score. Pearson correlation analysis was performed to assess associations between SP1 expression and xCell-derived immune infiltration scores. xCell results were reported because this algorithm provides enrichment scores across a broad range of immune and stromal cell types and was consistent with the immune-cell signatures emphasized in the present study.

### 2.9 Immune checkpoint gene expression profiling

The expression levels of key immune checkpoint genes were extracted from the log2(TPM + 1)-transformed TCGA ESCC expression matrix. The analyzed checkpoint genes included IGSF8, ITPRIPL1, SIGLEC15, TIGIT, CD274, HAVCR2, PDCD1, CTLA4, LAG3, and PDCD1LG2. ESCC samples were stratified according to SP1 expression, and checkpoint gene expression was compared between SP1-high and SP1-low groups.

### 2.10 TIDE-based predicted immunotherapy response analysis

To explore the association between SP1 expression and predicted immunotherapy-related features, Tumor Immune Dysfunction and Exclusion (TIDE) analysis (26) was performed using the public ESCC microarray dataset GSE22954 and GSE23400. Because the GPL8300 platform contained a larger tumor-only cohort suitable for quartile-based stratification, GPL8300 tumor samples were used for the main TIDE analysis. Probe-level expression values were collapsed to Entrez gene-level expression values, followed by log2(x + 1) transformation and per-gene sample centering before TIDE calculation. Tumor samples were ranked according to SP1 expression and divided into four quartile groups: Q1-low, Q2, Q3, and Q4-high. TIDE score, T-cell dysfunction score, T-cell exclusion score, interferon-gamma-related score, and predicted responder status were compared across SP1 expression quartiles. Continuous TIDE-related scores were compared using the Mann-Whitney U test for pairwise comparisons, and predicted responder proportions were compared using Fisher’s exact test.

### 2.11 Assessment of immunotherapy-treated ESCC datasets

We evaluated publicly available or publicly summarized transcriptomic data from immunotherapy-treated ESCC cohorts. In the ORIENT-2 sintilimab arm (27), pretreatment SP1 expression was analyzed as log2(TPM + 1) and compared between patients with objective response (complete or partial response) and those with stable or progressive disease using the Mann-Whitney U test. Progression-free and overall survival were compared after median SP1 dichotomization using log-rank tests. We also examined public post-treatment malignant-epithelial differential-expression results from Yang et al. (28)(BioProject PRJCA013236; HRA003591/HRA007035). GSE203115 and GSE289185 were reviewed for independent validation but were considered descriptive because the former included only three patients and the latter lacked patient-level response labels. The paired patient-level transcriptomic matrix associated with PRJCA013236 was under controlled access and was not available for analysis. No confirmatory biomarker inference was made from these underpowered or incompletely annotated datasets.

### 2.12 Cell culture

The human ESCC cell line EC109 was used for SP1 co-immunoprecipitation and mass spectrometry analysis. EC109 cells were cultured in RPMI-1640 medium supplemented with 10% fetal bovine serum and 1% penicillin-streptomycin at 37 °C in a humidified incubator containing 5% CO2. Cells were passaged at approximately 70% to 80% confluence and were used during the logarithmic growth phase for downstream experiments.

### 2.13 Co-immunoprecipitation of SP1

EC109 cells were lysed in RIPA buffer supplemented with protease inhibitors. Lysates were cleared by centrifugation, and protein concentration was quantified using the BCA assay. For each immunoprecipitation, 1 to 2 μg of anti-SP1 antibody or control IgG was incubated with protein A/G magnetic beads at 4 °C for 2 h with rotation. The antibody-coated beads were then incubated with 500 to 1,000 μg of total protein lysate overnight at 4 °C. After extensive washing with lysis buffer, the immunoprecipitated complexes were eluted.

### 2.14 LC-MS/MS analysis of SP1-co-immunoprecipitated proteins

Proteins co-immunoprecipitated with anti-SP1 antibody (21962-1-AP, Proteintech) from EC109 cells were subjected to in-gel digestion. Gel pieces were destained, dehydrated, and digested with trypsin (20 ng/μL in 50 mM NH4HCO3) at 37 °C for 16 h. The resulting peptides were extracted, lyophilized, and reconstituted in 0.1% formic acid before mass spectrometry analysis. Peptides were separated on an EASY-nLC 1200 system using a C18 analytical column with a gradient of mobile phase A (0.1% formic acid in water) and mobile phase B (0.1% formic acid in 80% acetonitrile). Mass spectrometric detection was performed on a Q Exactive HF-X mass spectrometer in positive ion mode with a full scan range of 300 to 1,400 m/z at a resolution of 120,000. The top 60 precursors per full scan were selected for higher-energy collisional dissociation fragmentation, and MS/MS spectra were acquired at a resolution of 7,500.

### 2.15 Functional enrichment and interaction analysis of SP1-binding partners

SP1-co-immunoprecipitated proteins identified by LC-MS/MS were ranked according to interaction abundance. The top-ranked proteins were submitted to Metascape for functional enrichment analysis. Enriched biological processes, cellular components, and pathway terms were summarized with emphasis on RNA metabolism, RNA splicing, cell-cycle regulation, and nuclear protein complexes. In addition, proteins associated with RNA splicing pathways were submitted to the STRING database to construct a protein-protein interaction network (29).

### 2.16 General statistical analysis

Statistical analyses were performed using R or Python. Spearman or Pearson correlation analysis was used as specified for each analysis. One-way analysis of variance followed by Tukey’s multiple-comparison test (which controls the family-wise error rate across all pairwise comparisons) was used to compare SP1 A.H.-scores across pathological stages in the 4NQO model. Given the small sample size (n = 3 per stage), the normality and homogeneity-of-variance assumptions underlying the ANOVA could not be formally verified; the corresponding results are therefore interpreted as descriptive, and the associated conclusions are appropriately qualified. The Mann-Whitney U test or Fisher’s exact test was used for TIDE-related group comparisons, as appropriate. For the treated-cohort assessment, the Mann-Whitney U test and log-rank test were used as described in Section 2.11. P values were adjusted using the Benjamini-Hochberg method when multiple-testing correction was required. A two-sided P < 0.05 was considered statistically significant. NS, not significant; *P < 0.05, **P < 0.01, ***P < 0.001, ****P < 0.0001.

## 3 Results

### 3.1 Single-cell omics identifies key transcription factors in esophageal epithelial inflammation-to-cancer transformation

Using the published CRA002118 epithelial transition-state dataset, we performed secondary analysis of stage-associated genes across the EpiC1-EpiC6 epithelial states, which range from normal and inflamed epithelium to invasive malignant states (**Figure 1A**). The heatmap in **Figure 1B** uses a zero-centered divergent color scale to better visualize stage-dependent gene expression differences across the EpiC1-EpiC6 states. TRRUST-based transcription factor enrichment analysis showed that SP1 increased in rank across the pairwise state comparisons and became the top-ranked transcription factor in the EpiC6-versus-EpiC5 comparison, exceeding classical inflammation-associated transcription factors such as NFKB1 and RELA (**Figure 1C**). The gene sets supporting the SP1 enrichment at the late transitions are shown as heatmaps of their cluster marker values across EpiC1-EpiC6 in **Figure 1C** (EpiC2→EpiC3, n = 33; EpiC3→EpiC5, n = 35; EpiC5→EpiC6, n = 24).

**Figure 1:**
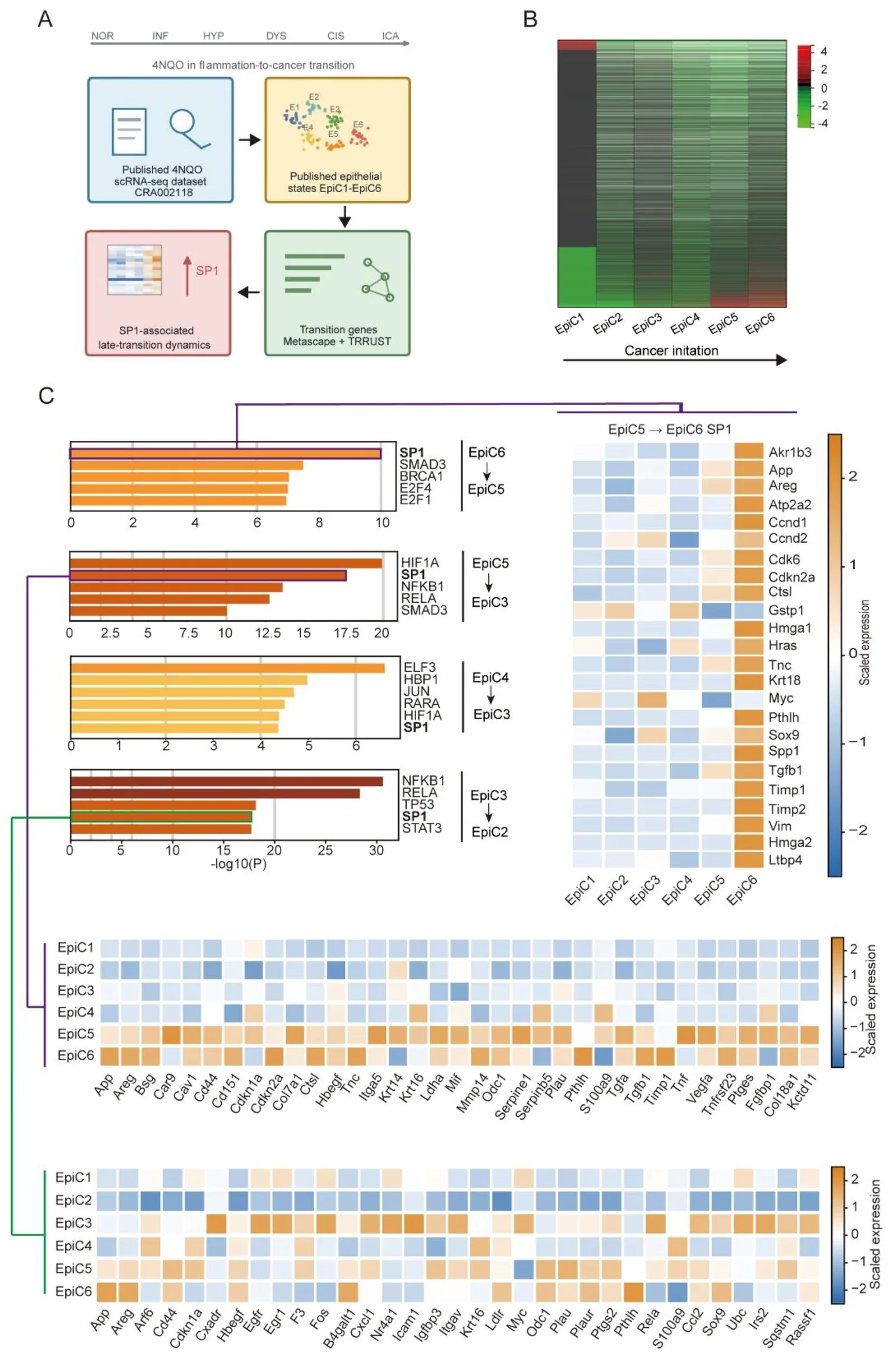
Transcription factor dynamics during inflammation-to-cancer transformation in esophageal epithelial cells. **(A)** Secondary-analysis workflow based on the published processed 4NQO single-cell epithelial transition-state dataset. **(B)** Global heatmap of stage-associated gene-expression patterns across EpiC1-EpiC6, shown using a zero-centered divergent color scale. **(C)** TRRUST transcription factor enrichment for the five pairwise epithelial state comparisons (EpiC2 vs EpiC1, EpiC3 vs EpiC2, EpiC4 vs EpiC3, EpiC5 vs EpiC3 and EpiC6 vs EpiC5; left); the EpiC2 vs EpiC1 comparison yielded no significantly enriched transcription-factor cluster and is therefore not plotted.

### 3.2 SP1 protein expression gradually increases during the inflammation-to-cancer transformation of ESCC

We next examined SP1 protein expression using the 4NQO-induced spontaneous esophageal carcinogenesis mouse model. Representative immunohistochemistry showed weak nuclear SP1 staining in normal epithelium, whereas staining increased across inflammation, hyperplasia, dysplasia, carcinoma *in situ*, and invasive carcinoma (**Figure 2A**). Consistently, the nuclear A.H.-score (mean ± SD) was 61 ± 10, 99 ± 8, 139 ± 11, 161 ± 3, 191 ± 9, and 229 ± 9 across the same sequence (**Figure 2B**). These quantitative values show a stage-associated increase in SP1 protein expression during inflammation-to-cancer transformation.

**Figure 2:**
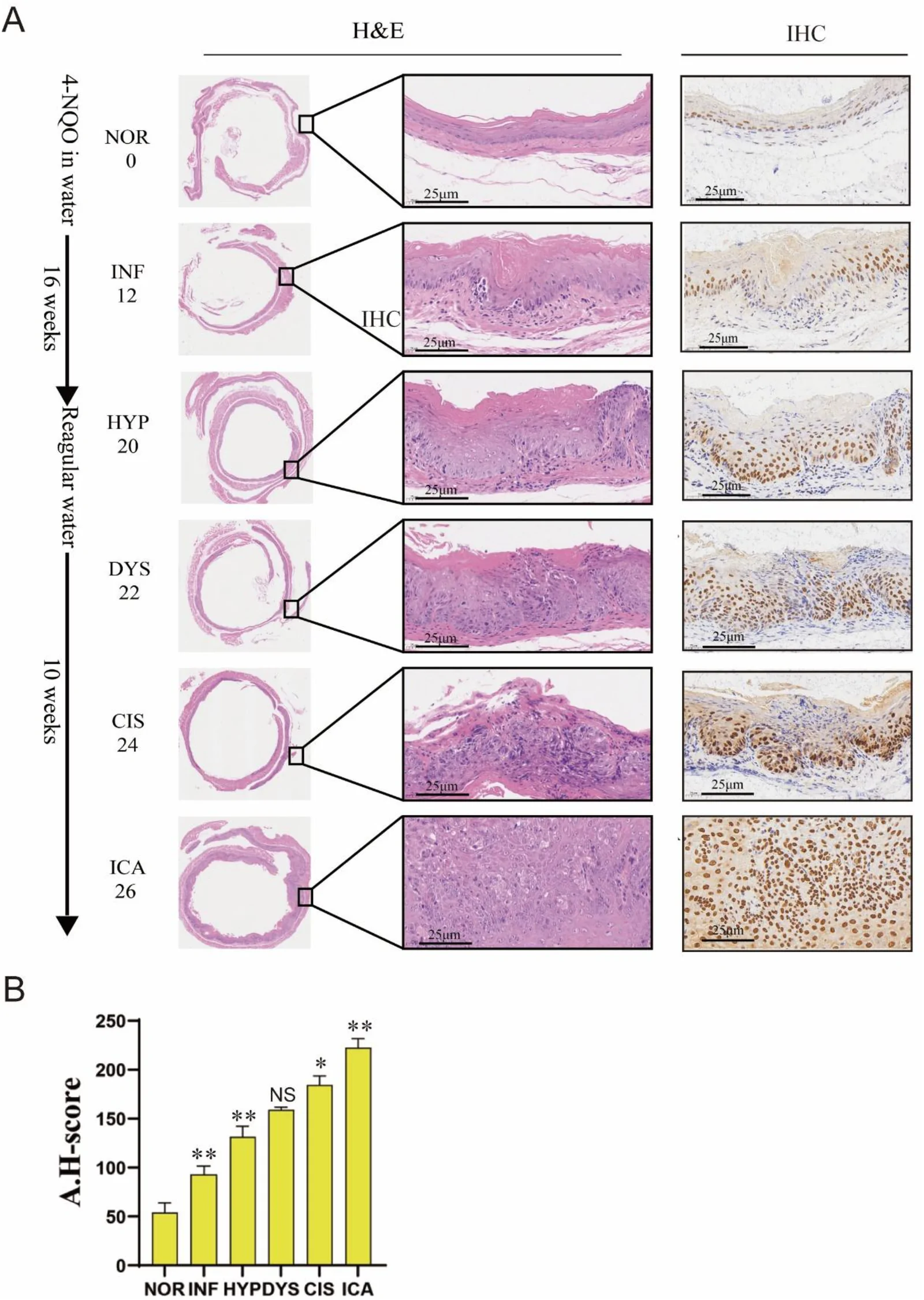
SP1 protein expression during inflammation-to-cancer transformation in mouse esophageal epithelium. **(A)** Representative H&E staining and SP1 immunohistochemistry of esophageal tissues from mice treated with 4NQO for 12, 20, 22, 24, and 26 weeks. **(B)** Nuclear A.H.-score (H-score) across pathological stages (n = 3 independent animals per pathological stage), range from 0 to 300, and are presented as mean ± SD. Statistical significance was evaluated by one-way ANOVA followed by Tukey’s multiple-comparison test. NS, not significant; *P < 0.05 and **P < 0.01 versus the immediately preceding stage.

### 3.3 Context-dependent virtual SP1 perturbation reveals inflammation-associated epithelial remodeling and tumor-state regulatory programs

Using scTenifoldNet and scTenifoldKnk, we simulated SP1 overexpression and knockout in epithelial cells from adjacent normal esophageal tissue and ESCC tissue (**Figures 3A, D**). In adjacent normal epithelial cells, SP1 overexpression mainly perturbed S100A8, S100A9, IL1RN, STMN1, GSTM3, HMGN5, CSTA, KRT6B, TOP2A, KRT15, CRABP2, TUBB, and MT1X, whereas SP1 knockout affected genes including STMN1, KRT14, GSTM3, SPRR3, and MT2A (**Figures 3B, C**). In ESCC epithelial cells, SP1 overexpression mainly influenced CNFN, CCL5, LY6D, TNFSF9, KRT15, FNBP1, and CSTB, whereas SP1 knockout affected SAMD9, DSP, REL, PIK3R3, GLUL, and FGFBP1 (**Figures 3E, F**).

**Figure 3:**
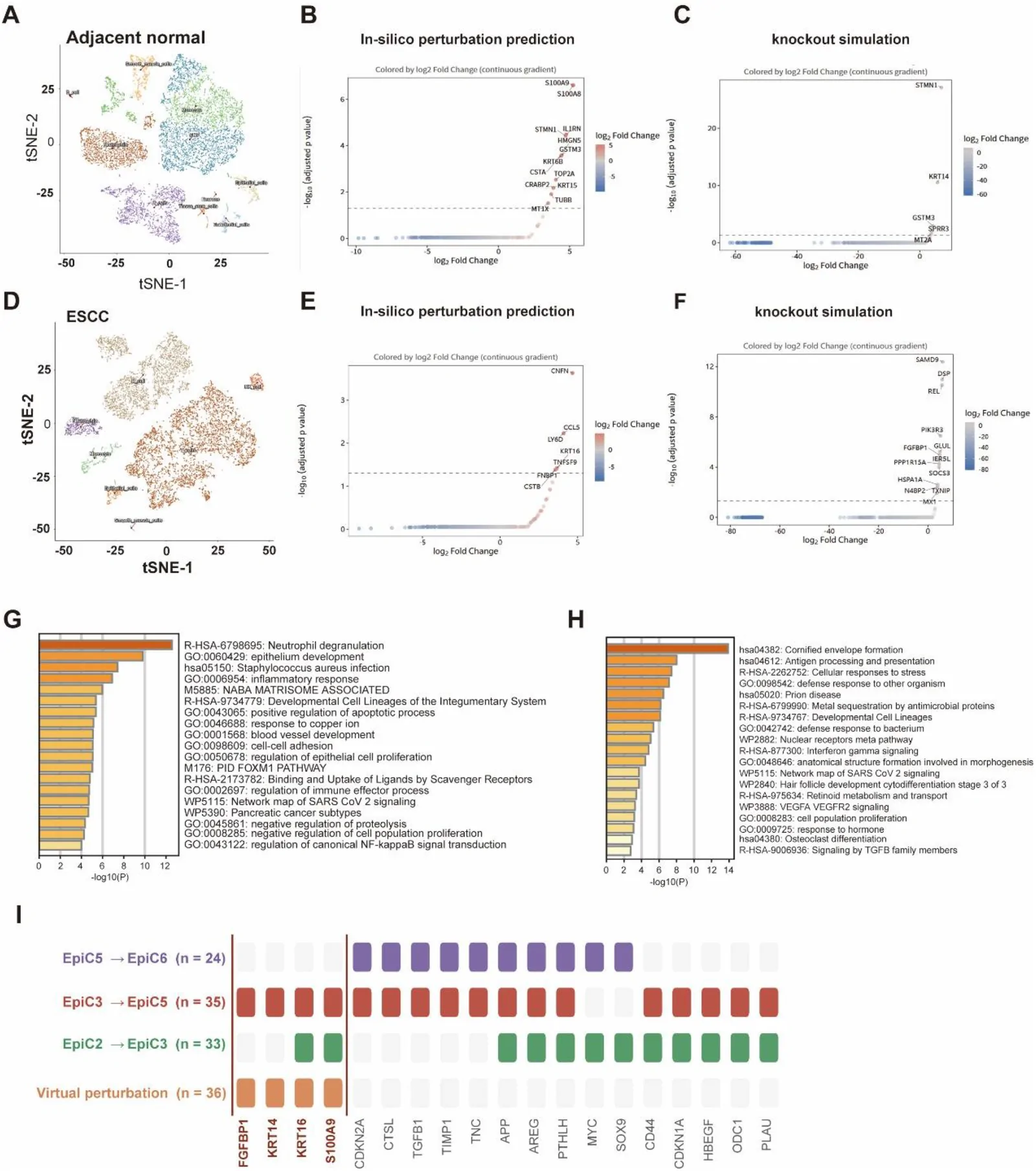
Virtual SP1 perturbation analysis in epithelial cells from adjacent normal esophageal tissue and ESCC tissue. **(A)** Dimensionality-reduction visualization of adjacent-normal epithelial cells. **(B, C)** Volcano plots showing genes perturbed by simulated SP1 overexpression and knockout, respectively, in adjacent-normal epithelial cells. **(D)** Dimensionality-reduction visualization of ESCC epithelial cells. **(E, F)** Volcano plots showing genes perturbed by simulated SP1 overexpression and knockout, respectively, in ESCC epithelial cells. **(G, H)** Functional enrichment of the top 100 perturbed genes following SP1 overexpression in adjacent-normal epithelial cells and SP1 knockout in ESCC epithelial cells, respectively. **(I)** Overlap between the TRRUST-SP1-supporting gene sets of the three late epithelial transitions of the corrected trajectory and the genes significantly affected by virtual SP1 perturbation in normal epithelium or ESCC cells; filled squares indicate membership of the corresponding gene set.

Functional enrichment of the top 100 perturbed genes further showed that SP1 overexpression in adjacent normal epithelial cells was associated with inflammatory response, neutrophil degranulation, NF-κB signaling, epithelial development, epithelial proliferation, cell-cell adhesion, and stress-related programs (**Figure 3G**). By contrast, SP1 knockout in ESCC epithelial cells was enriched for cornified envelope formation, antigen processing and presentation, interferon-gamma signaling, VEGFA-VEGFR2 signaling, collagen-fibril assembly, and TGF-beta family signaling (**Figure 3H**). Because all analyses were restricted to epithelial cells, immune-related terms were interpreted as epithelial-cell-intrinsic inflammation-associated programs rather than direct immune-cell activity.

To determine which step of the corrected epithelial trajectory is most closely linked to SP1-dependent regulation, we compared the TRRUST-SP1-supporting genes of its late transitions (EpiC2→EpiC3, EpiC3→EpiC5 and EpiC5→EpiC6) with genes significantly affected by virtual SP1 perturbation in normal epithelium or ESCC cells (**Figure 3I**). The EpiC3→EpiC5 gene set was significantly enriched for SP1-perturbation-responsive genes (4 of the 13 genes assessable in the perturbation background: KRT14, S100A9, KRT16, and FGFBP1; hypergeometric P = 0.0013), whereas none of the assessable EpiC5→EpiC6 genes overlapped this set. The same perturbation-responsive genes also intersected the EpiC2→EpiC3 transition (n = 33), which shared KRT16 and S100A9 with the EpiC3→EpiC5 set (2 of 16 assessable genes), whereas FGFBP1 and KRT14 were specific to the EpiC3→EpiC5 transition; neither KRT16 nor S100A9 was present in the EpiC5→EpiC6 set. The overlap with the virtual SP1 perturbation is therefore concentrated in the EpiC2→EpiC3 and EpiC3→EpiC5 transitions and is not maintained at the later EpiC5→EpiC6 stage.

### 3.4 SP1 virtual perturbation genes are associated with poor prognosis in ESCC

Based on the SP1 virtual perturbation genes derived from ESCC epithelial cells, we performed an exploratory LASSO regression analysis to evaluate the relationship between these genes and tumor prognosis in the TCGA ESCC cohort. The final risk score formula was established as follows: Risk score = (-0.0487)*PIK3R3+(0.0707)*GLUL+(-0.0088)*IER5L+(0.2653)*PPP1R15A+(-0.4232)*N4BP2+(0.2134)*TXNIP, with λ min = 0.04.

Gene coefficients, partial likelihood deviance, individual risk scores, survival status, and gene expression levels are presented in **Figures 4A–E**. After dichotomizing patients into high-risk and low-risk groups according to the median risk score, a significant prognostic difference was observed between the two groups, with median overall survival of 1.7 years in the high-risk group and 3.5 years in the low-risk group (P = 0.000849; **Figures 4F, G**). The 3-year area under the curve (AUC) of the model was 0.879. Because this model was developed and evaluated within the same TCGA-ESCC cohort and lacks independent external validation, the 3-year AUC of 0.879 should be interpreted as exploratory.

**Figure 4:**
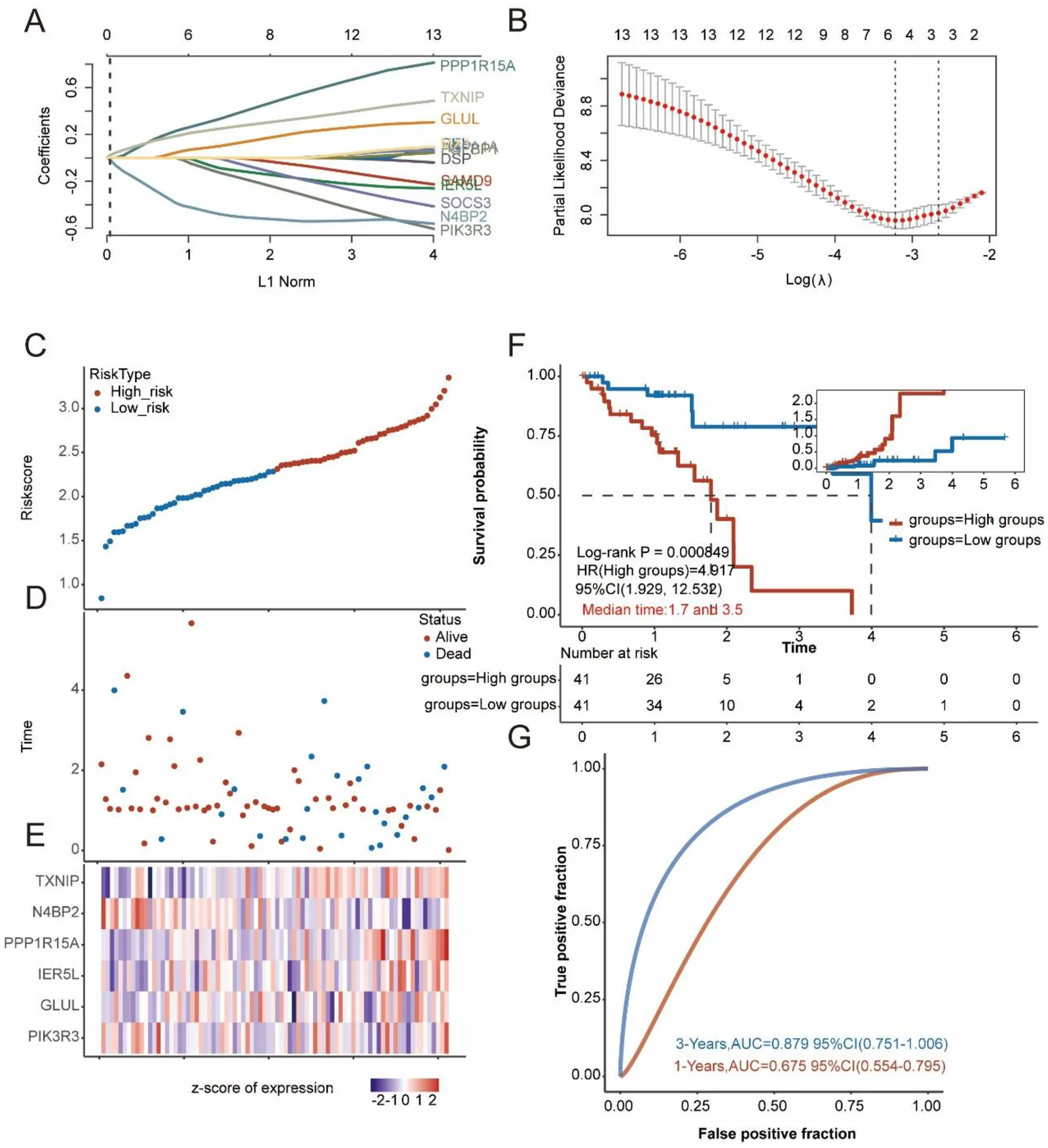
Exploratory prognostic model based on SP1 perturbation genes. **(A–E)** Gene coefficients, partial likelihood deviance, individual risk scores, survival status, and gene expression levels. **(F, G)** Prognostic performance and Kaplan-Meier survival analysis of the risk-score model. The model was developed and evaluated within the same TCGA-ESCC cohort without independent external validation; its results should be interpreted as exploratory.

### 3.5 SP1-associated pathway programs in ESCC

To further investigate SP1-associated pathway programs in ESCC, we performed single-sample gene-set enrichment scoring and correlation analysis using TCGA ESCC samples. SP1 expression was positively correlated with lysine degradation, inositol phosphate metabolism, the G2M checkpoint, TGF-beta signaling, cysteine and methionine metabolism, and biosynthesis of unsaturated fatty acids, and was negatively correlated with amino sugar and nucleotide sugar metabolism and oxidative phosphorylation (**Figures 5A–I**).

**Figure 5:**
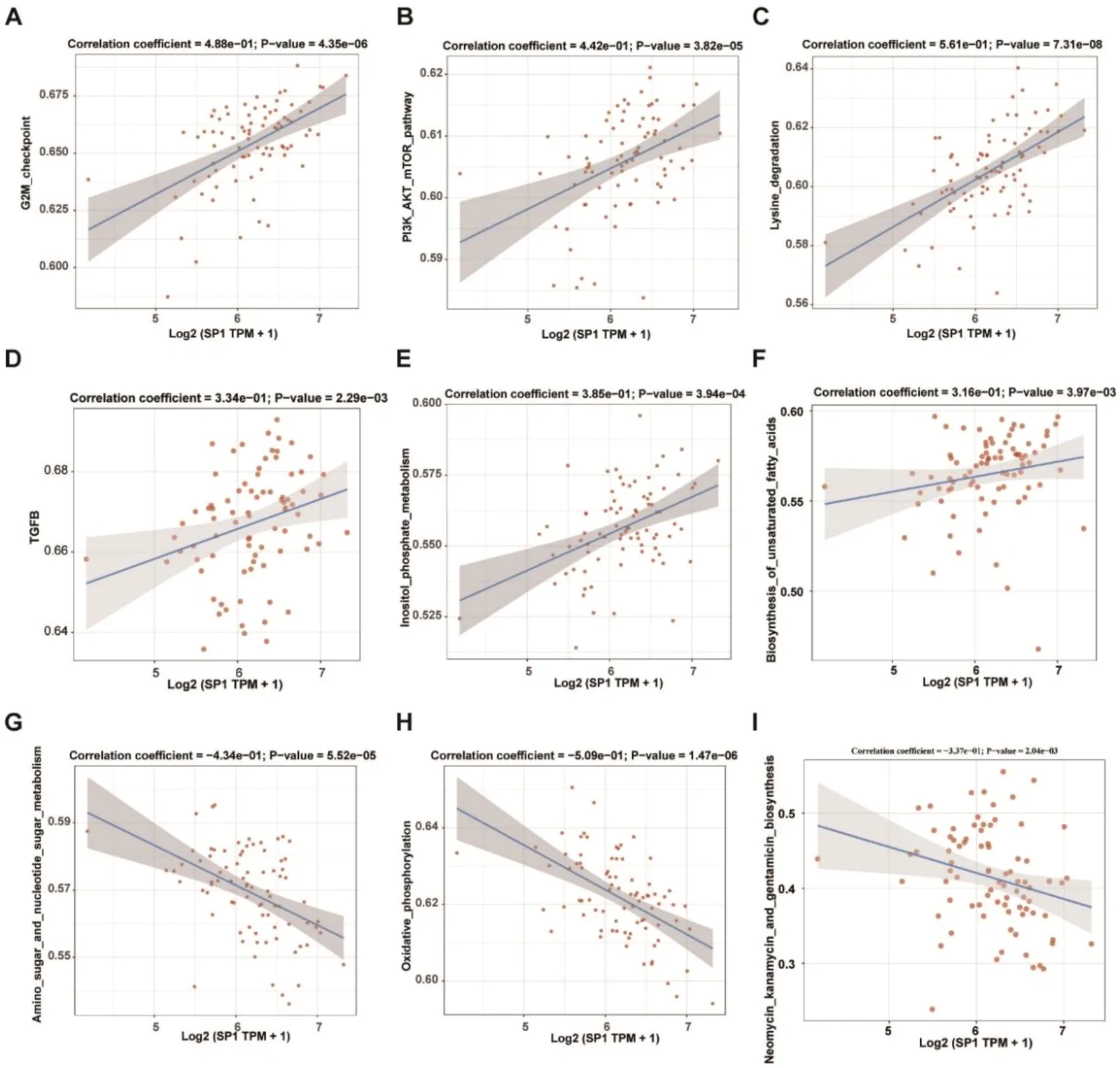
**(A–I)** Pathway correlation analysis of SP1-associated cellular programs in TCGA ESCC samples. Panels show representative pathways whose single-sample gene-set enrichment scores were significantly correlated with SP1 expression.

### 3.6 Association between SP1 and immune infiltration features

Using the xCell algorithm, we assessed immune infiltration signatures correlated with SP1 expression based on TCGA ESCC data. Monocytes, M2 macrophages, CD4+ Th1 cells, CD4+ effector memory T cells, and the immune score showed significant negative correlations with SP1 expression, whereas CD4+ memory T cells showed a significant positive correlation (**Figures 6A–F**). Correlations among the infiltrating immune-cell signatures are shown in **Figure 6G**.

**Figure 6:**
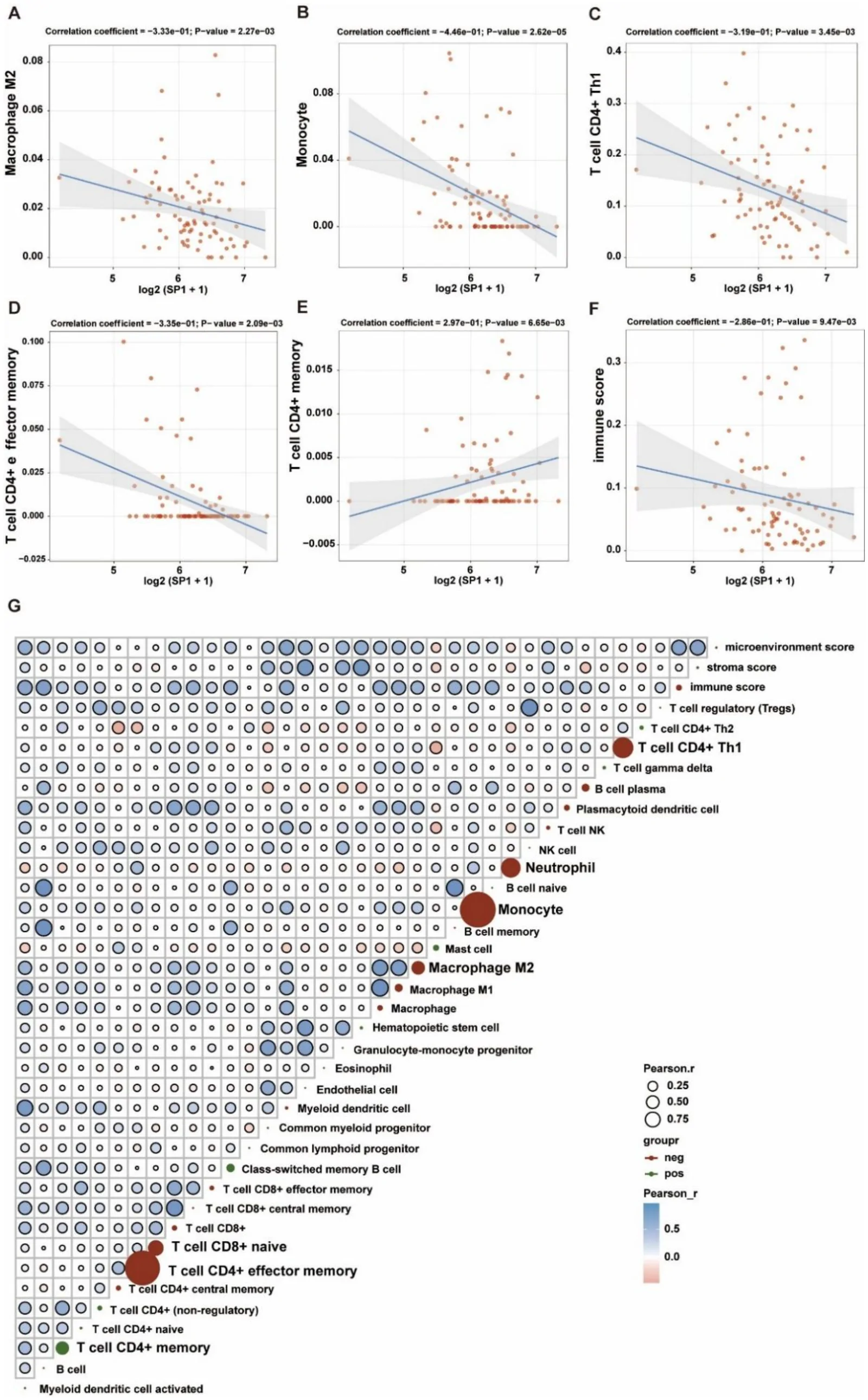
xCell analysis of SP1-associated immune infiltration features in ESCC samples. **(A–F)** xCell-derived immune-cell or immune-score signatures significantly correlated with SP1 expression. **(G)** Correlation matrix among the significantly associated immune infiltration signatures.

### 3.7 Association between SP1, immune checkpoint genes, and TIDE-predicted immunotherapy-related features

Given the association between SP1 and immune infiltration, we examined the relationship between SP1 expression and immune checkpoint genes in TCGA ESCC samples. Several immune checkpoint genes, including CTLA4, ITPRIPL1, PDCD1, SIGLEC15, and TIGIT, showed higher expression in the SP1-high group (**Figure 7A**). We further performed TIDE-based exploratory analysis to evaluate the association between SP1 expression and predicted immunotherapy response. In the GSE22954_GPL8300 and GSE23400_GPL96 tumor-only cohort, samples were divided into four SP1-expression quartiles (Q1-low, Q2, Q3, and Q4-high). Compared with Q1-low tumors, Q4-high tumors showed lower TIDE scores (p = 7.41 × 10–^5^ and p = 0.012) and a higher predicted responder proportion (p=0.000244 and p = 0.054) (**Figures 7B, C**). These findings suggest that SP1 expression is associated with immunotherapy-related immune features; however, because TIDE provides computational prediction rather than direct evidence from an immunotherapy-treated ESCC cohort, the immunotherapy-related interpretation remains exploratory.

**Figure 7:**
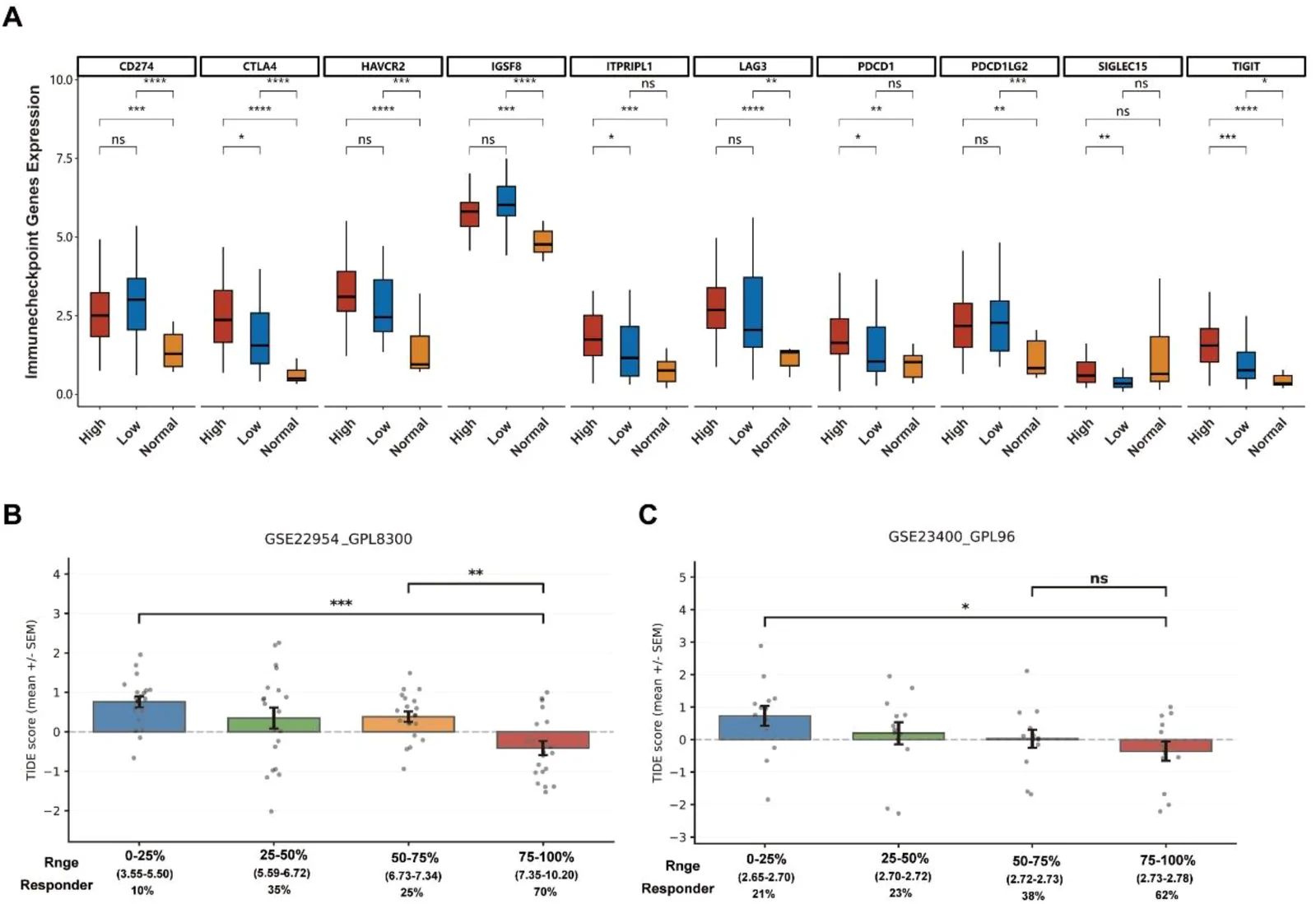
Association between SP1 expression, immune checkpoint genes, and TIDE-based predicted immunotherapy-related features in ESCC. **(A)** Expression of immune checkpoint genes in SP1-high, SP1-low, and normal tissue groups. **(B, C)** TIDE-based comparison of predicted immunotherapy-related scores and predicted responder status according to quartile-based SP1 expression grouping. * P<0.05, **P<0.01, ***P<0.001, ****P<0.0001, ns, no significance.

### 3.8 Identification of candidate SP1-associated protein networks in ESCC cells

Using co-immunoprecipitation coupled with HPLC-MS/MS, we identified candidate SP1-associated proteins in EC109 ESCC cells (**Figure 8A**). Enrichment analysis showed that these proteins were annotated to mitotic cell-cycle regulation, membraneless organelle assembly, and regulation of RNA splicing (**Figure 8B**). STRING analysis further illustrated the interaction network of RNA-splicing-associated proteins among the SP1-binding candidates (**Figure 8C**), and subcellular localization enrichment mapped part of the protein set to spliceosome- and methyltransferase-complex-related annotations (**Figure 8D**). These findings suggest that SP1 may be associated with RNA-processing-related protein networks in ESCC cells; however, Co-IP/MS enrichment alone does not demonstrate direct post-transcriptional regulation by SP1.

**Figure 8:**
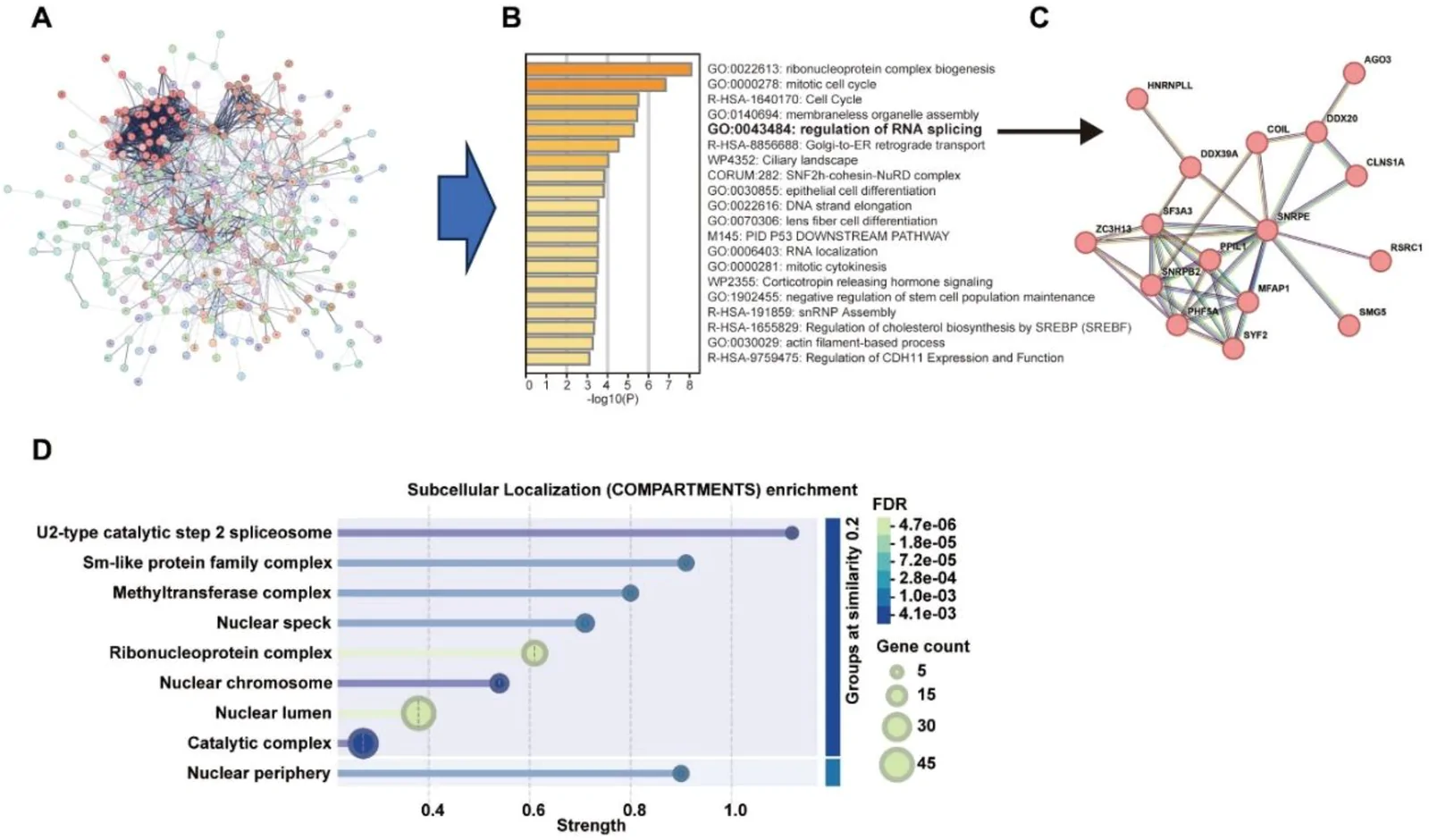
Bioinformatic analysis of candidate SP1-associated proteins identified by Co-IP/MS in EC109 ESCC cells. **(A)** Interaction network of binding partner proteins identified by HPLC-MS/MS. **(B)** Metascape clustering of the top 200 proteins ranked by interaction abundance. **(C)** STRING network analysis of proteins associated with the RNA splicing pathway among the binding partners. **(D)** Subcellular localization enrichment of SP1-associated proteins. These analyses indicate potential associations between SP1-binding candidates and RNA-processing-related protein networks, rather than experimentally proving post-transcriptional regulation by SP1.

## 4 Discussion

Our study integrates multi-level evidence from single-cell omics, transcription factor activity, virtual perturbation, immune infiltration, prognostic prediction, and protein–protein interaction mass spectrometry to investigate the role of SP1 in esophageal inflammation-to-cancer transformation. Our results support a model in which SP1 becomes progressively more prominent across the pairwise epithelial state comparisons and is associated with state-dependent regulatory programs.

Through single-cell omics analysis, we revealed that SP1 exhibits an increasing enrichment trend across the epithelial state comparisons and becomes the top-ranked transcription factor in the EpiC6-versus-EpiC5 comparison, and ranks above classical inflammation-associated factors such as NFKB1 and RELA in the preceding EpiC5-versus-EpiC3 comparison (**Figure 1**). This finding suggests that as the esophageal epithelium progresses from an inflammatory state to a malignant state, the center of the regulatory network may undergo a shift. In normal epithelium, SP1 primarily influences genes such as KRT14, SPRR3 (30), GSTM3, and STMN1, indicating its potential involvement in basal cell maintenance, squamous differentiation, oxidative stress response, and epithelial renewal (31). Moreover, overexpression of SP1 also affects inflammation-related genes including S100A8, S100A9, and IL1RN, suggesting that its aberrant upregulation is associated with a state of inflammatory activation and stress-induced remodeling in normal epithelium (32, 33). In contrast, in cancer cells, virtual SP1 knockout predominantly impacted genes such as SAMD9, DSP, REL, PIK3R3, GLUL, and FGFBP1, whereas virtual SP1 overexpression mainly influenced CCL5, TNFSF9, KRT15, and related genes; these genes have been associated with inflammatory signaling, tumor cell survival, immune modulation, and a stem/progenitor-like state (34–37). This pattern is consistent with the interpretation that, in the context of malignancy, the function of SP1 is no longer confined to maintaining epithelial homeostasis; instead, it may be co-opted by tumor cells, its regulatory network may undergo state-dependent remodeling and is associated with malignant proliferation, immune microenvironment regulation, and cell fate reprogramming.

Along the corrected trajectory, in which EpiC4 represents a differentiation branch rather than a step in malignant progression, the SP1-dependent regulatory program was most clearly linked to the EpiC3→EpiC5 transition. The subsequent EpiC5→EpiC6 program shared 8 genes with the EpiC3→EpiC5 set but was not enriched for perturbation-responsive genes, suggesting that it represents a downstream expansion of the SP1-associated network rather than a further increase in direct SP1 dependence. Among these candidates, S100A9 is of particular interest. This interpretation is supported by SP1 occupancy at the S100A9 promoter and by keratinocyte data linking SP1 activity to S100A8/A9 expression (38, 39). Functionally, S100A8/A9 may translate this program into tumor-promoting inflammation by driving Akt/p38-dependent invasion in ESCC and RAGE/NF-κB signaling and myeloid immunosuppression in other tumors (32, 40, 41). Thus, S100A9, together with KRT14, KRT16, and FGFBP1, may connect SP1 activity with inflammation-driven ESCC progression, although direct transcriptional regulation remains to be established.

Classic studies have established that SP1 extensively participates in and finely regulates gene expression through transcription-related mechanisms. SP1 specifically binds to GC-rich sequences in DNA (e.g., the GC box 5’-GGGCGG-3’) via its three C-terminal C2H2 zinc finger domains and recruits transcriptional coactivators (such as CBP/p300) and the basal transcriptional machinery (such as the TFIID complex) through its N-terminal transactivation domains, thereby initiating the transcription of target genes (42). In ESCC, however, SP1 exhibits a “transcriptional inertness” phenotype: data from the TCGA database and single-cell sequencing of the Linzhou ESCC cohort in Henan Province have confirmed that its mRNA expression is not significantly upregulated and its transcriptional activity is weak (43). These seemingly conflicting observations may be reconciled if SP1 abundance and activity in human ESCC are regulated at the protein or post-translational level rather than at the mRNA level; however, direct measurement of SP1 protein in human ESCC tissues is still needed to support this interpretation.

In recent years, several studies have reported the RNA-binding capacity of SP1. Jingwen Song et al. found in HEK293T cells that SP1 recognizes and binds G-rich sequences in mRNA 3′UTRs to regulate alternative splicing (44). Further studies have revealed that this canonical C2H2 zinc finger transcription factor can directly bind RNA in living cells, preferentially targeting the single-stranded loop regions of short RNA stem-loops, and that its RNA-binding ability antagonizes its chromatin DNA-binding function and participates in the post-transcriptional regulation of alternative polyadenylation of target mRNAs (45). It has also been shown that the RNA-binding regions of SP1 are concentrated in m^6^A-modified enriched 3′UTRs (45), which is consistent with our detection of SP1 interactions with RNA splicing-related proteins. Additionally, SP1 has been reported to promote cancer progression through a phase separation mechanism (46). It is thus speculated that during the inflammation-to-cancer transformation of the esophageal squamous epithelium, the regulatory network of SP1 may undergo a major shift, transitioning from purely transcriptional regulation toward a mode that may integrate both transcriptional and post-transcriptional regulation; direct experimental evidence (e.g., RNA-IP, splicing assays, or phase-separation experiments) is required to substantiate this possibility. Because the present Co-IP/MS analysis did not identify an inflammation-specific interaction program, we regard it as an exploratory observation of candidate RNA-processing associations rather than evidence for the inflammation-to-cancer axis.

The transcription factor SP1 has been implicated in the transcriptional regulation of immune checkpoints. Previous studies have demonstrated that SP1 can directly bind to the SP1 element in the PD-L1 promoter and cooperate with ZEB2 to transcriptionally activate PD-L1 expression (20). SP1 also forms a transcriptional complex with YAP/TEAD4 to positively regulate VISTA expression (21). In prostate cancer, SP1 has been identified as a key transcription factor that drives B7-H3 expression induced by PTEN and TP53 deficiency (22). In cervical cancer, targeting SP1 reprograms histone modifications and consequently modulates the expression of immune checkpoint genes (47). These findings suggest that SP1 may function as a pan-cancer transcriptional regulatory hub for immune checkpoints. Consistently, in ESCC, high SP1 expression correlates with the upregulation of multiple immune checkpoint molecules, including TIGIT, SIGLEC15, PDCD1, ITPRIPL1, and CTLA4 (**Figure 7**), which is consistent with but does not directly demonstrate, direct transcriptional activation of these checkpoint genes by SP1; promoter-binding evidence (e.g., ChIP-seq or promoter-reporter assays) is required to establish causality. Of note, CD274 (PD-L1), a well-documented SP1 transcriptional target in other cancer types, was not among the significantly upregulated checkpoint genes in the present ESCC cohort; this discrepancy warrants cautious interpretation and may reflect cohort-specific or context-dependent regulation.

Available treated-cohort data did not reproduce the TIDE-predicted direction. In ORIENT-2, only 4 of 56 response-evaluable sintilimab-treated patients responded, and SP1 was associated with neither response nor survival. In the public post-treatment epithelial comparison from Yang et al., SP1 was lower in responders, whereas the other available datasets were either too small or lacked patient-level response labels. These discordant and underpowered observations do not support SP1 as a standalone immunotherapy-response biomarker; rather, the TIDE association should be viewed as a hypothesis about immune context that requires validation in a sufficiently powered pretreatment cohort.

Several limitations should be acknowledged. The virtual perturbation, TIDE, and immune-correlation analyses are computational and require experimental validation (e.g., SP1 knockdown/overexpression with transcriptomic profiling, and ICB-treated cohorts). The Co-IP/MS experiment was performed in a single cell line without independent validation, and direct evidence for non-canonical RNA-related functions of SP1 (e.g., ChIP-seq, RNA-IP, or phase-separation assays) is still lacking. SP1 protein expression was validated only in the mouse model, with adjacent normal tissue from ESCC patients as the human single-cell reference, and the prognostic model was based on a small TCGA-ESCC cohort without external validation. Our findings should therefore be regarded as hypothesis-generating.

In summary, SP1 emerged as a progressively prominent transcription factor across the epithelial state comparisons spanning inflammation-to-cancer progression, and cross-species integration prioritized S100A9, together with KRT14, KRT16, and FGFBP1, as candidate mediators of an SP1-associated epithelial program. These results support SP1 as a candidate regulatory hub but do not establish it as an immunotherapy-response biomarker or therapeutic target. Future studies should test direct SP1-dependent regulation and the proposed RNA-related associations using adequately powered human cohorts and functional perturbation experiments.

## Data Availability

The 4NQO single-cell epithelial transition-state dataset (CRA002118; GSA, https://ngdc.cncb.ac.cn/gsa/); human ESCC single-cell RNA-sequencing dataset (GSE196756); ESCC microarray datasets used for TIDE analysis (GSE22954 and GSE23400); and datasets reviewed for descriptive comparison (GSE203115 and GSE289185), all from the Gene Expression Omnibus (GEO; https://www.ncbi.nlm.nih.gov/geo/); TCGA-ESCA data (https://portal.gdc.cancer.gov/). The raw mass-spectrometry proteomics data underlying the SP1 Co-IP/LC-MS/MS analyses have been deposited to the ProteomeXchange Consortium (https://proteomecentral.proteomexchange.org) via the iProX partner repository(48, 49) with the dataset identifier PXD084403. The custom analysis scripts used in this study are available from the corresponding author on reasonable request.
